# Comparative analysis of low complexity regions in Plasmodia

**DOI:** 10.1038/s41598-017-18695-y

**Published:** 2018-01-10

**Authors:** S. R. Chaudhry, N. Lwin, D. Phelan, A. A. Escalante, F. U. Battistuzzi

**Affiliations:** 1 Department of Biological Sciences, Oakland University, Rochester, MI USA; 2 Center for Molecular Medicine and Genetics, Wayne State University, Detroit, MI USA; 3 Institute for Genomics and Evolutionary Medicine, Temple University, Philadelphia, PA USA; 4 Center for Data Science and Big Data Analytics, Oakland University, Rochester, MI USA

## Abstract

Low complexity regions (LCRs) are a common feature shared by many genomes, but their evolutionary and functional significance remains mostly unknown. At the core of the uncertainty is a poor understanding of the mechanisms that regulate their retention in genomes, whether driven by natural selection or neutral evolution. Applying a comparative approach of LCRs to multiple strains and species is a powerful approach to identify patterns of conservation in these regions. Using this method, we investigate the evolutionary history of LCRs in the genus Plasmodium based on orthologous protein coding genes shared by 11 species and strains from primate and rodent-infecting pathogens. We find multiple lines of evidence in support of natural selection as a major evolutionary force shaping the composition and conservation of LCRs through time and signatures that their evolutionary paths are species specific. Our findings add a comparative analysis perspective to the debate on the evolution of LCRs and harness the power of sequence comparisons to identify potential functionally important LCR candidates.

## Introduction

Regions within sequences that are composed of a lower diversity of residues (nucleotides or amino acids) compared to other areas can be defined as low complexity regions (LCRs). These regions can have many different configurations, from repetitive single amino acids (also known as homorepeats, single amino acid repeats, or homopolymeric regions; here abbreviated as HPRs) to aperiodic motifs of multiple residues, and are present in coding and non-coding areas of a genome^1–3^. Within coding regions, many studies have shown the widespread presence of LCRs in eukaryotes and prokaryotes with percentages of proteins with at least one LCR ranging from less than 10% to more than 50%^1,4–6^. Recently, comparative analyses of LCRs within protein-coding areas have started delineating some of their fundamental properties, which include species-specific compositions, high evolutionary rates of these regions and their flanks, and a generally low conservation across strains and species^7–9^. However, despite a growing body of information, mechanisms that regulate the formation and retention of LCRs over evolutionary times are still poorly understood^10^.

Multiple studies have shown the importance of LCRs in phenotypic plasticity with some suggesting an active role of positive and purifying selection in their evolution^10–15^. Others, instead, promote neutral evolution as a primary mechanism for the observed changes across species and strains^5,8,9,16–19^. The potential association of these regions with phenotypic plasticity makes them a particularly interesting target to investigate the origin of pathogenicity and the evolution of host-pathogen interactions, especially in species, like *Plasmodium falciparum*, that are known for their high frequency of LCRs^5,7,10,14,20–22^. However, only the LCRs in the genomes of few Plasmodium species have been analyzed and comparative studies are mostly focused on multiple strains of the same species (e.g.^7,23^) or specific gene families across a few strains and species (e.g.^20,24^). Therefore, an analysis of complete proteomes of multiple Plasmodium species and strains will provide a more comprehensive picture of LCR evolution over time.

To achieve this, we investigated LCRs in protein-coding regions of Plasmodia for four primary reasons: *first*, the use of protein-coding LCRs allows an evolutionary study of LCRs present in orthologous genes; *second*, the high frequency of LCRs in *Plasmodium falciparum* provides a large comparative baseline to determine the evolution of these regions in other Plasmodia species; *third*, the range of compositional biases in different Plasmodia (from AT-rich to AT-balanced) is a useful property to test the effect of genome compositional biases on LCRs evolution; *fourth*, the availability of fully sequenced genomes of multiple species and strains of Plasmodia provides the necessary information for a long-term evolutionary study. The species we are comparing include four primate-infecting plasmodia, *P. vivax* (strains Salvador, India, North Korea, Brazil, and Mauritania; *Pvs, Pvi, Pvnk, Pvb, Pvm*), *P. cynomolgi* (*Pcy*), and *P. knowlesi* (*Pk*) (collectively called the CVK group) and *Plasmodium falciparum* (*Pf*), which is the most studied genome within the Plasmodium genus. These comparisons allow the exploration of evolutionary changes over ~50 million years of evolution and among species with different genome biases and host preferences^21,25–27^. Species of the CVK group are of particular interest because they form a closely related clade that includes one of the two primary human pathogens (*Pv* and *Pf*) in addition to *Pcy* and *Pk* that have been recently reported as the cause of several other human malaria cases^28–35^. Because the hosts of *Pcy* and *Pk* are primarily macaques, comparative genomics of these species with preferentially human-host plasmodia can provide insights into potentially host-specific evolutionary strategies and recent or ongoing adaptive changes.

Using comparative genomics and ancestral state reconstruction methods we found unique compositional trends in LCRs of different species that are unrelated to their proteome compositional biases. We also show that there is a strong tendency for low complexity regions of a specific type (either HPRs or LCRs) to remain the same over evolutionary time suggesting a slow turnover of one type into the other. For those regions that change type over time, we find a similar frequency from HPR to LCR and *vice versa*. Overall, these results suggest a role for non-neutral mechanisms driving the evolution of LCRs in Plasmodia.

## Results

The identification of LCRs and HPRs is a complex process that requires the specification of at least two parameters: the minimum size of the region to be identified and a threshold that determines the difference between high and low complexity areas^21^. Using HPRs instead of LCRs is advantageous because it eliminates the necessity of defining a complexity threshold, since the only requirement for a HPR is to have a single repetitive amino acid (in these analyses we used a window size of 6^21,36^). For this reason, we use HPRs as our starting point in the following analyses. This is a conservative approach that, while reducing the amount of information available, restricts the regions analyzed to those that can be more objectively identified.

### Compositional analyses

**Figure 1:**
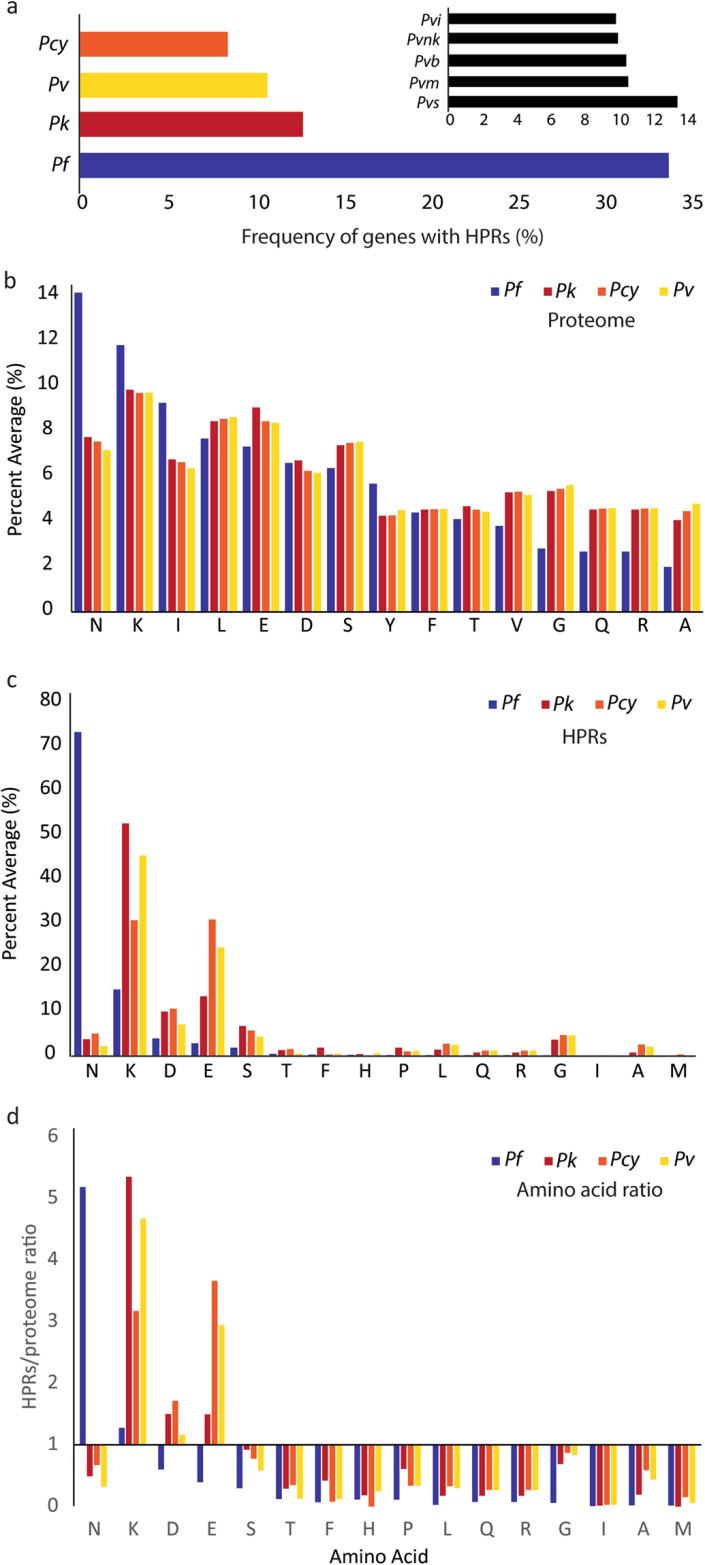
Frequency and composition of homopolymeric regions (HPRs) in *Plasmodium*. (**a**) Frequency of HPRs in *P. falciparum* (*Pf*), *P. vivax* (*Pv*), *P. cynomolgi* (*Pcy*), and *P. knowlesi* (*Pk*) (CVK group). Frequencies are calculated as the ratio of proteins with ≥1 HPRs and the total number of proteins in each species. The inset shows the frequency of HPRs for 5 *Pv* strains (*Pvb: P. vivax Brazil I; Pvi: P. vivax India VII; Pvm: P. vivax Mauritania; Pvnk: P. vivax North Korea; Pvs: P. vivax Salvador-1*). (**b**) and (**c**) usage of amino acids in the proteome (**b**) and the HPRs (**c**) of *Pf* and the CVK group. (**d**) Ratio of amino acid usage in HPRs and proteomes. Only amino acids with >1% frequency in any of the species are shown in decreasing order of frequency in *Pf*.

Genomes of the CVK group are AT-rich (~70%) but exomes are more balanced (50–60%)^26^, in contrast with *Pf* where both genome and exome are AT-rich (>75%). Following expectations from compositional biases of these species, and in agreement with previous studies, we have found that *Pf* has a high frequency of HPRs (34%) but that this frequency is reduced by approximately two thirds in the CVK group (Fig. 1a). Within the five *Pv* strains, *Pvs* has the highest frequency of HPRs (~13%) while the other strains are lower and similar to each other (~10%) (Fig. 1a inset).

**Figure 2:**
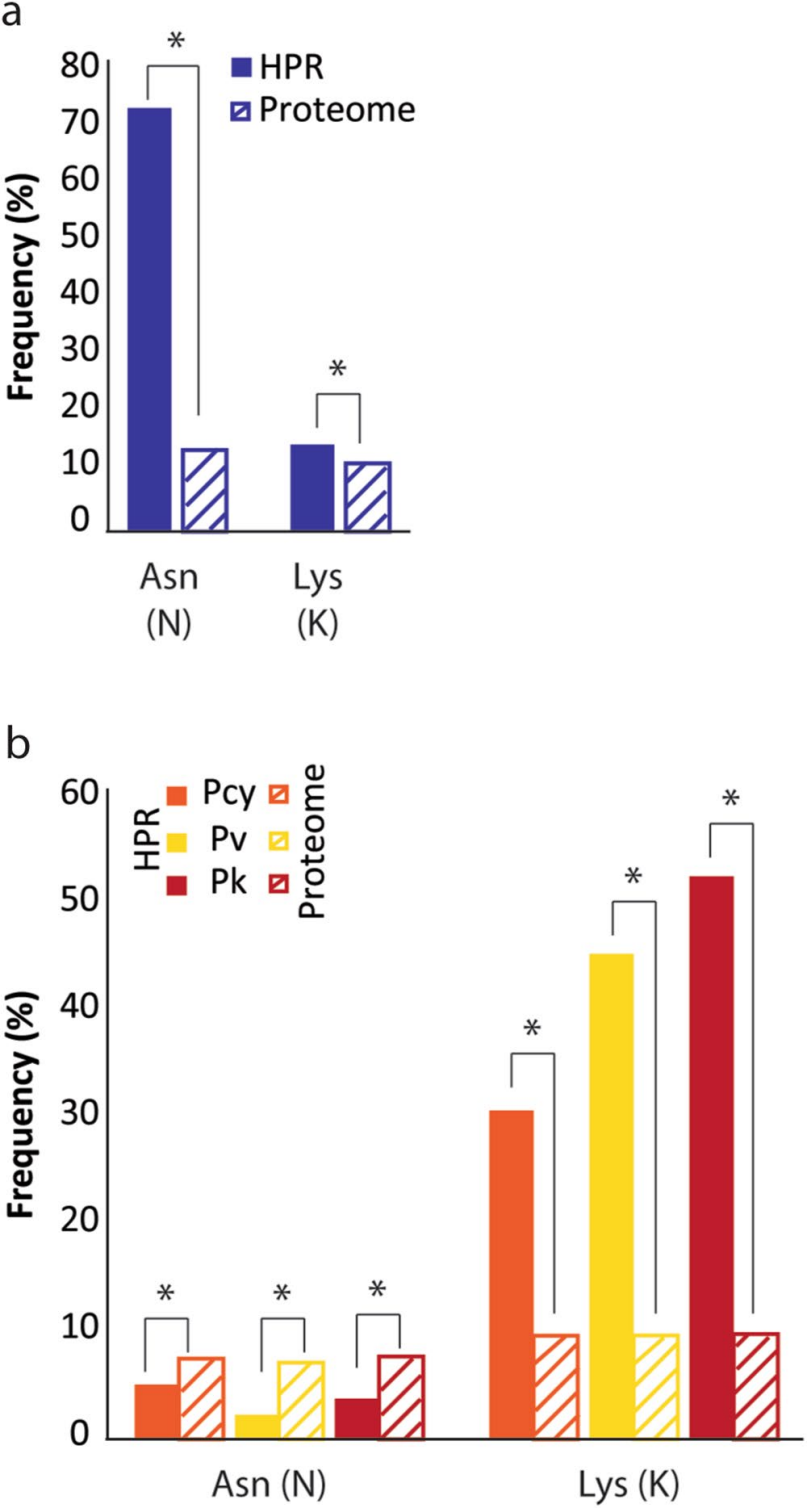
Asparagine and Lysine usage in HPRs and proteome of *Pf* (**a**) and the CVK lineages (**b**). Asterisks show significant t-test p-values at the 1% level. Additional statistical t-tests not shown are: HPRs *Pf* Asn vs. CVK Asn and *Pf* Lys vs. CVK Lys, both significant at p ≪ 0.001.

A compositional analysis of these regions shows a significant difference in amino acid usage in *Pf* compared to the CVK group (Fig. 1c) with a strong preference for Asparagine (Asn, N) in *Pf* (73.4%) and for Lysine (Lys, K) in the CKV group (31–53%)^7,37^. Within the CVK group, *Pv* and *Pcy* also show 25–31% usage of Glutamic acid (Glu, E), which constitutes <5% of the HPRs amino acids in *Pf* (usage of Glu in *Pk* is also lower at ~12%). Of these amino acids, the AT-richness of Lys is in contrast with the mostly balanced compositional preference of the CVK exomes while the usage of Asn in *Pf* (high-AT) and Glu in *Pcy* and *Pv* (balanced-AT) follows the AT-composition of these genomes and exomes. Although the two most preferred amino acids within HPRs are also the most frequently used in the proteome of *Pf* and CVK (Fig. 1b), this trend does not extend to other amino acids commonly used in the proteome. For example, in *Pf* Ile is used in the proteome with a similar frequency to that of Asn and Lys but its presence in HPRs is less than 1%. In the CVK group multiple amino acids are used with similar frequencies in the proteome (Lys, Glu, Leu: 8–10%) but only Lys and Glu are significantly used (>10%) in HPRs. Overall, comparisons of amino acid usage between proteome and HPRs show strong differences with Asn 5-fold more frequent in *Pf* HPRs compared to the proteome (73% *vs*. 14%) and Lys at least 3 times more frequent in CVK HPRs *vs*. proteome (31–53% *vs*. 10%) (Figs 1d and 2). These results suggest that proteome and HPR compositions are not directly correlated and that other forces contribute to the composition of HPRs.

We also compared the length of HPRs among species in relation to the length of proteins in which the repeats were found. On average, the length of these regions is similar in *Pf* and CVK (~7.5 amino acids), which is close to the minimum number of amino acids (6) used to identify HPRs (see Methods). This length is unrelated to the size of the proteins hosting HPRs (p-values > 0.05 for each species), suggesting that HPR length is not an emergent property of protein size (Supplementary Fig. S1). Interestingly, in the CVK group ~75% of the proteins that contain HPRs contain more than one, while in *Pf* ~33% of the proteins have multiple HPRs. This is not surprising given the higher frequency of HPRs in *Pf* compared to CVK.

### Evolutionary analyses

To determine the evolutionary history of HPRs in the CVK group, we identified single-copy orthologs present in all species and extracted those that had HPRs in at least one of the species, resulting in a total of 846 orthologs. We then divided them in three categories based on their relative conservation levels:

(i) Full conservation (FC): HPRs are identical in length and composition in all 7 lineages.
(ii) *P*. *vivax* conservation (VC): HPRs are identical among the five *Pv* strains but can vary in length and/or composition in *Pcy* and *Pk*.
(iii) No conservation (NC): HPRs that are not conserved in either length or composition, or are conserved in lineages that do not share a most recent common ancestor (e.g., conserved in *Pv* and *Pk* but not in *Pcy*).

As expected from the fast evolutionary rates of these regions^7,38^, a majority (72%; 605) of the identified orthologous HPRs belong to the NC category, while 25% (212) and 3% (29) are in the VC and FC category, respectively. The two conserved categories have partially overlapping compositional preferences with both having a strong preference for Lys (65.4% and 40.7% in FC and VC, respectively) followed by Leu in FC (14.3%) and Glu in VC (16.3%) (Supplementary Fig. S2). Overall, HPRs of the VC category are composed of 13 amino acids (K > E > S > G > D > L > A > N > Q > P > R > F > H) while those in FC use 5 amino acids (K > L > E > S > I). These trends, however, should be interpreted with caution given the small number of genes especially in the FC category.

To characterize the nature of the conserved HPRs, we performed Gene Ontology (GO) enrichment analyses of *Pvs* full proteome, the 846 *Pvs* sequences belonging to the orthologous genes, and the genes containing the FC and VC HPRs independently. While the proteome showed no enrichment for any functional category, the 846 orthologs, the FC, and the VC groups were statistically significantly enriched for 27, 26, and 26 categories across molecular function, cellular function, and biological processes, respectively (Fisher’s exact test p-value < 0.05 and false discovery rate <0.05; see Supplementary Table S1 and Fig. S3). If we discard GO IDs that are present in less than 10 genes the numbers decrease to 22, 22, and 10 respectively. The represented functional categories vary and include both house-keeping (e.g., transcription) and metabolic functions. Most of the categories are unique to each gene subset with very few shared by multiple sets. For example, the categories shared by the 846 orthologs and the VC group include cell structure-related functions, such as cytoskeleton and microtubule-based process; the categories shared by the 846 group and the FC group are cell and biological adhesion. Interestingly, the two conserved HPRs categories (FC and VC) do not share similar functions, suggesting that the probability of conservation of an LCR is unrelated to the function of its gene.

**Figure 3:**
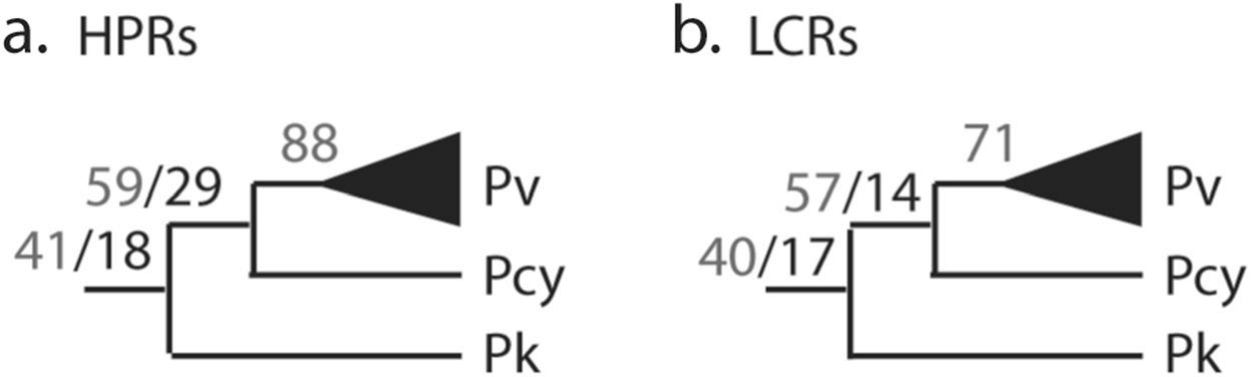
Ancestrally reconstructed low complexity regions (LCRs) in the CVK group. Results are shown for regions conserved in the *Pv* strains as HPRs (**a**) or LCRs (**b**); in these cases, these regions are present as homopolymers in at least one of the other species). From tips to root, numbers in grey represent conserved states (HPR → HPR or LCR → LCR) and numbers in black represent changes (HPR → LCR or LCR → HPR).

We also used a subset of the VC low complexity regions (159 HPRs in any of the CVK species; see Methods) to determine their evolutionary history by ancestral state reconstruction. This subset represented those VC genes with a topology in agreement with current knowledge *((Pv, Pcy), Pk)* and also obtained by a consensus phylogenetic analysis of five random samples of 100 orthologous genes each^39,40^. Based on this topology, we reconstructed the most likely evolution of HPRs at each ancestral node to determine the probability of changes between HPRs and LCRs. Our results show a similar trend in HPRs and LCRs with most of these regions remaining of the same type in the CVK group throughout evolutionary time and only 20–33% changing from one type to the other at each ancestral node (Fig. 3). Additionally, we found that the changes that led to a shift from HPRs to LCRs or *vice versa* are more likely among amino acids of similar structure and size and are unrelated to the frequency of the changing amino acids in the proteome. For example, the two most common amino acid changes, which explain 51% of the substitutions identified, are Lysine (K) ↔ Arginine (R) (first and eleventh most frequently used amino acids in the *Pv* proteomes, respectively) and Aspartic Acid (D) ↔ Glutamic Acid (E) (seventh and second most frequently used amino acids in the *Pv* proteomes, respectively). In the first pair, Lys and Arg, have a positively charged side chain while in the second pair, Asp and Glu, have a negatively charged side chain. This result suggests that these substitutions are more likely to be retained by evolution when they have minimal disruptive effects on the structure of the protein rather than being neutrally driven by amino acid usage in the proteomes.

## Discussion

The role played by low complexity regions within Plasmodium species is debated. Evidence from previous studies have supported either a neutral or functional role of these regions based on experimental and computational analyses of regions primarily in *Plasmodium falciparum* and few other selected species and strains^10,20,23,41–43^. A more comprehensive evolutionary approach has the potential to clarify the role played by LCRs in relation to phenotypic variability by adding information on the history of these regions through time. Here, we have used a comparative genomics approach of orthologous proteins in 7 Plasmodium lineages to reconstruct compositional preferences and evolutionary histories of their LCRs.

**Figure 4:**
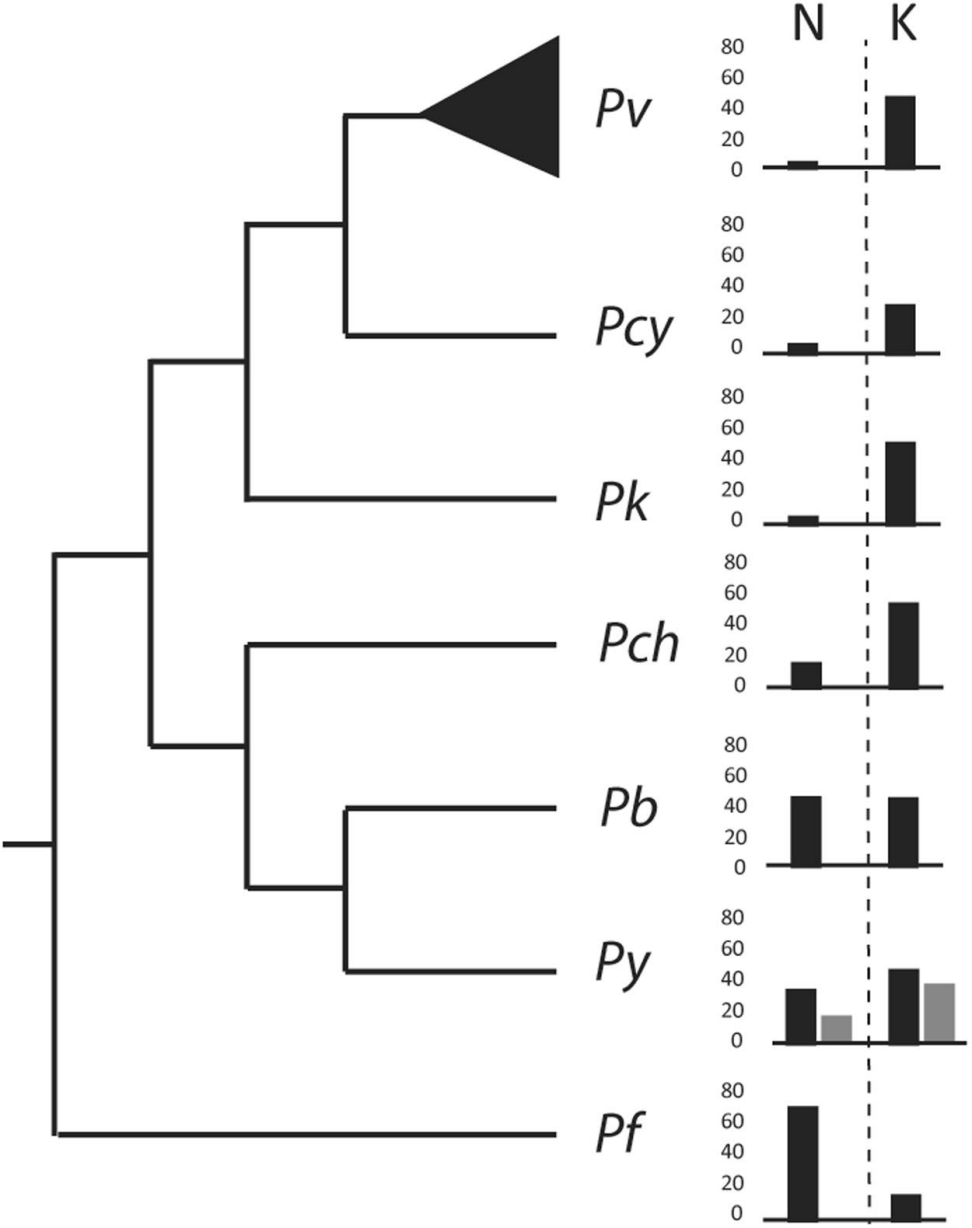
Preferred amino acid usage in HPRs of the Plasmodium genus. Values for *Pv* are averages of the 5 strains. *Py* includes values calculated for strains *17X* (darker shade) and *YM* (lighter shade).

We found a significant difference in the composition of HPRs between *Pf* and the CVK group. *Pf* has a strong preferential use of Asn (Asparagine, N) while the CVK group more frequently uses Lys (Lysine, K) (Fig. 2). A compositional bias towards AT richness in the genome of *Pf* has previously been suggested as a potential cause for the widespread use of Asn in LCRs^44^, implying that these regions evolve neutrally. Our results do not support this explanation for three reasons: *first*, the two most commonly used amino acids in *Pf* are Asn and Lys but Asn is 6-times more frequent than Lys. Being that both are encoded by AT-rich codons (5/6 nucleotides are A or T), compositional bias alone cannot explain the preferential selection of one over the other. *Second*, Lys is preferentially used in the CVK group that is substantially less AT-rich than *Pf*. *Third*, we analyzed the compositional preference of three rodent-infecting plasmodia (*P. berghei, P. yoelii*, and *P. chabaudi*) that are AT-rich like *Pf*. Contrary to *Pf*, these species preferentially use Lys instead of Asn in their low complexity regions, further supporting our hypothesis that amino acid preference in LCRs is not driven by genome-wide biases (Fig. 4). Past studies have proposed other mechanisms, beyond compositional bias, to explain the composition of LCRs. For example, Cruz *et al*.^45^ suggested that hydrophobic and acidic amino acids are more likely to be used in LCRs compared to amino acids belonging to other Lehninger categories. However, our results do not show this preference as Asn and Lys belong to the “polar uncharged” and “basic” categories, respectively. Alternatively, replication slippage and non-homologous recombination^19,26^ could also bias the composition of LCRs. Previous studies have correlated the strength of replication slippage with codon usage preferences but also highlighted the importance of selection in maintaining the amino acid purity of a repeat^46^. While an investigation of LCRs at the nucleotide level will provide information on the potential strength of replication slippage based on codon usage preferences for each amino acid, codon usage alone is unlikely to be able to explain the preferential choice of Asn over Lys in *Pf* (or *vice versa* in the other species) as these two amino acids are encoded, obviously, by different sets of codons. Therefore, we suggest that our results are best explained by a role of selection determining the amino acid composition of these regions with a possible later role of replication slippage in their expansion and contraction.

The importance of the composition of low complexity regions becomes apparent when considering their potential functional role. The role of Asn-rich regions in Pf is unknown but past research in *Pf* has shown that exported proteins are often Lys-rich and that their function can be disrupted if the length and/or composition of these regions is altered (reviewed in^10^). It is possible that Lys-rich regions in the CVK group may play similar functional roles and, indeed, we have found that genes that are conserved across CVK lineages are preferentially located to the transmembrane cellular compartment (Supplementary Fig. S3 and Table S1). Similarly, these genes are often associated with binding and adhesion functions, although this is not exclusive to conserved HPRs. The observed functional category enrichments suggest a possible role of selection in the conservation of Lys-rich residues to maintain their localization signature and function. In pathogenic species such as *Plasmodium*, exported and transmembrane gene products are expected to be involved in host-pathogen interactions. The presence of low complexity regions in genes involved in host-pathogen communication is known in other organisms such as viruses suggesting the possibility of a previously undetected role of LCRs in the evolution of pathogenic strategies^7,47^.

Another indication of a potential functional role of conserved LCRs in Plasmodium comes from an ancestral state reconstruction analysis that allowed us to model the evolutionary path of these regions over the approximately 10 million years of evolution since the ancestor of the CVK group^48^. Irrespective of their ancestral state with one or more amino acids (HPR or LCR), we found that most of these regions (70–80%) remains unaltered in type (in other words, it remains an HPR or an LCR as it was ancestrally). This result is unexpected under a neutral (non-functional) model of evolution for these regions since the purity of a region is expected to decrease with time (thus predicting a higher transition rate of HPRs into LCRs) in the absence of mechanisms to explicitly maintain it^19,46^.

Taken together, these results provide multiple lines of evidence that suggest that selection is an important driving force in the evolution of Plasmodium LCRs over millions of years. While additional analyses will reveal the nature of these selective forces, this evidence is in agreement with a recent study that showed positive and weak purifying selection acting on repetitive regions in numerous species across the three domains of life^12^. Additionally, based on the species-specific signatures of LCRs that we identified, we also suggest that these selective forces are not driven by vector or host specificity of the pathogens but rather by each species independent evolution. In the case of the vector, the promiscuity of Plasmodium species in infecting the genus Anopheles means that there is no single selective pressure acting on each Plasmodium species; thus, any adaptive strategy in Plasmodium cannot be directly correlated to a specific vector species^33,49,50^. In the case of the host, comparisons of trends and patterns in LCRs between the two human plasmodia (*Pf* and *Pv*) show different frequencies and compositional preferences, which is likely the result of the two independent host shift events that allowed these species to colonize humans from other primates^51^. The implications of these independent evolutionary paths are potentially far-reaching as they suggest that these two species (and their strains) have different pools of phenotypic variability (i.e., different LCRs) to tap into for evolution and adaptation strategies, which could hinder the development of a single anti-malaria approach that would work globally^35,52,53^.

## Methods

### Data Collection

Proteomes of seven plasmodium lineages (*P. vivax Salvador-1, P. vivax India VII, P. vivax Brazil I, P. vivax Mauritania I, and P. vivax North Korea, P. cynomolgi strain B, and P. knowlesi strain H*, collectively called the CVK group) were retrieved from NCBI in addition to the proteome of *P. falciparum 3D7*. We also retrieved the proteome of *P. chabaudi* from PlasmoDB 11.0 that we used as outgroup for the phylogenetic analyses of the CVK group and for the compositional analyses of the rodent-infecting Plasmodia along with *P. yoelii yoelii* and *P. berghei* (also retrieved from PlasmoDB)^54^. We then derived two sets of orthologs, one for the comparative analysis of repetitive regions, which does not include the outgroup in order to maximize the number of orthologs identified, and one for the ancestral state reconstruction analysis in which the outgroup was included to produce a rooted phylogeny. All orthologs were identified with OrthoMCL using default parameters^55^ and only those groups with single copy genes present in the CVK lineages (and the outgroup if included) were analyzed. We then identified homopolymeric regions in each of these orthologs using the program SEG with the following parameters: window size of 6, determined empirically^21,36^, and complexity thresholds of 0 (K_1_ and K_2_)^56^. These parameters ensure that the minimum length of the regions is 6 amino acids and that they are formed by a single amino acid.

For each of the two ortholog categories (with and without outgroup) we obtained alignments with Muscle using default values^57^. Because repetitive regions, such as HPRs, are known to be difficult to align, all further HPRs-related analyses (i.e., conservation, functional, and ancestral state reconstruction analyses) were performed on well-conserved orthologs (see §Conservation of HPRs). Phylogenetic analyses, instead, were performed on five randomized subsets of 100 single-copy orthologs with the estimation of the best substitution models for each subset from ProtTest 3.3^58^ and the calculation of Maximum Likelihood phylogenetic trees in MEGA6^59^ using 1000 bootstrap replicates. The consensus phylogeny was then used as a reference to estimate the ancestral states of the HPRs (see §Ancestral State Reconstruction). Alignments and accession numbers of genes used are available upon request.

### Conservation of HPrs

For every HPR in the 4,148 orthologous groups identified, a pairwise comparison was done to determine the conservation level of these regions among species. To achieve this, we developed in-house a comparative algorithm that identifies HPR locations within an alignment, analyzes their flanking regions (defined as the 10 amino acids upstream and downstream of the HPR), and compares all sequences within the identified regions. In brief, the algorithm starts from the location of the HPRs identified by SEG and searches for other HPRs within the alignment that are partially overlapping. If found, the boundaries of the most upstream and most downstream HPR are used to define a new window with a larger repetitive region that will be further analyzed for its conservation. Combined HPR windows with gapped flanking regions were excluded from further analyses to avoid noise in the evolutionary signal caused by poor alignments. Conservation was determined based on the presence/absence of HPRs in all or some of the lineages and amino acid substitutions or gaps in pairwise comparisons of the HPRs. Based on this comparative analysis, we identified three levels of conservation: full conservation (FC), *P*. *vivax* conservation (VC), and no conservation (NC). Within the VC category, we identified two subgroups: one where the strains of *Pv* contain HPRs (and *Pcy* and *Pk* may contain LCRs) and another where the strains of *Pv* contain LCRs and the HPRs is present in either *Pcy* or *Pk* or both. These two categories were both used for the ancestral state reconstruction analysis (see below).

### Functional analysis of Proteins Containing HPRs

Using PlasmoDB, we identified the functional category for each protein containing HPRs based on Gene Ontology (GO) classifications^60^. We then conducted enrichment analyses using the GO enrichment tool in PlasmoDB on four datasets from *P. vivax Salvador I*: the full proteome, the 846 orthologous proteins containing HPRs, and the orthologs containing HPRs categorized in groups FC (29) and VC (212). Enrichment was evaluated at the 5% confidence level for the Fisher’s exact test p-value and the false discovery rate. Revigo was then used to create semantic similarity plots using default values and the *Plasmodium falciparum* database for GO term sizes^61^.

### Ancestral State Reconstruction of HPRs

Using five randomized subsets of 100 orthologs each (Kuo *et al*. 2008), we estimated the phylogenetic tree of the 8 Plasmodia (7 primate-infecting Plasmodia and one rodent-infecting plasmodium outgroup) in our study using MEGA6^59^. We then used the obtained consensus tree as a guide tree to estimate ancestral states of HPRs in PrographMSA^62^. We focused on those HPRs that were classified in the VC group and that produced in PrographMSA the same topology obtained with the consensus tree (159 total HPRs). Then, at each ancestral node, HPRs were categorized based on the type of change, if any, from LCR to HPR or *vice versa*.

### Data availability

The source data analysed during the current study are available in the PlasmoDB repository (plasmodb.org) and in NCBI (ncbi.nlm.nih.gov). Alignments, LCRs, and other analyses (e.g., compositional conservation of LCRs) are available from the corresponding author upon request.

## Electronic supplementary material

Supplementary material
