# Supplementary material for: Comparative analysis of low complexity regions in Plasmodia
Source: Sci Rep. 2018 Jan 10;8:335. doi: 10.1038/s41598-017-18695-y (PMC5762703; doi:10.1038/s41598-017-18695-y)
Supplement: Supplementary file 1 — Supplementary material [file 41598_2017_18695_MOESM1_ESM.pdf]

Supplementary Information

Comparative analysis of low complexity regions in Plasmodia

Chaudhry S[1,2], Lwin N[1], Phelan D[1], Escalante AA[3], Battistuzzi FU[1,*]

[1] Department of Biological Sciences, Oakland University, Rochester, MI

[2] Center for Molecular Medicine and Genetics, Wayne State University, Detroit, MI

[3] Institute for Genomics and Evolutionary Medicine, Temple University, Philadelphia, PA

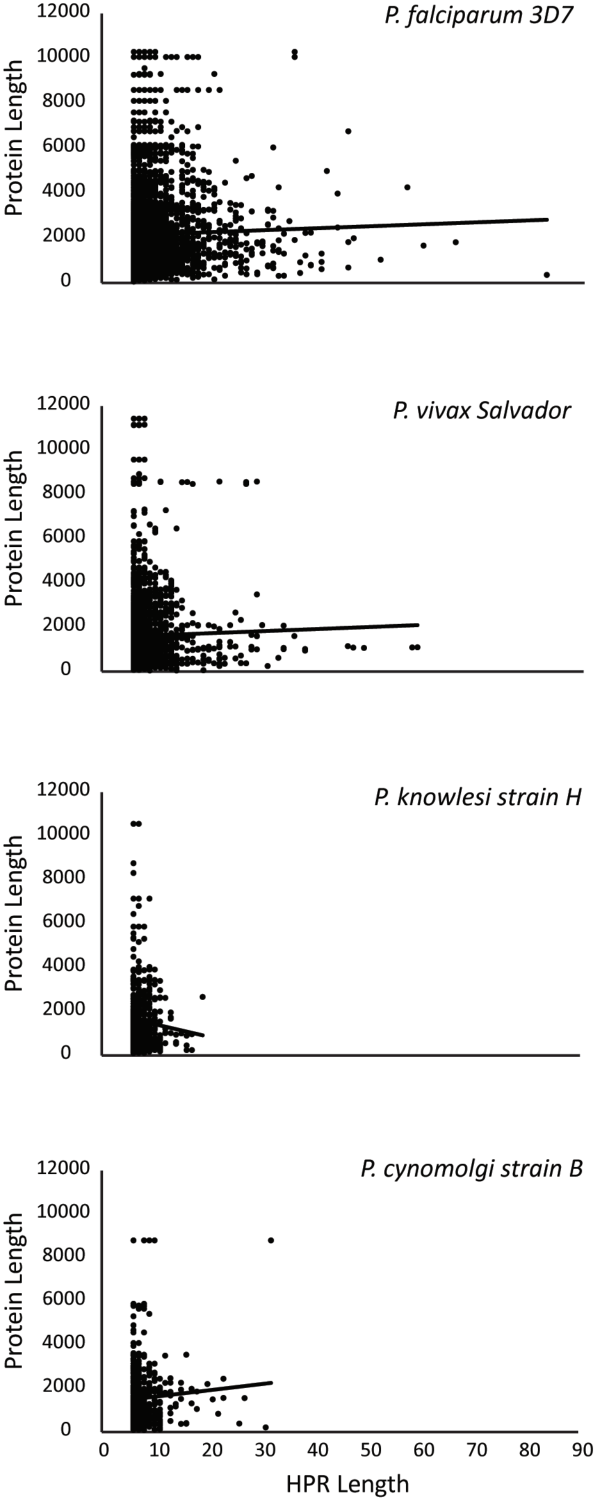

Fig. S1

Figure S1 Relationship of proteins and HPR lengths for four Plasmodium species. HPRs minimum length was set at 6 amino acids. Associated trendlines are shown.

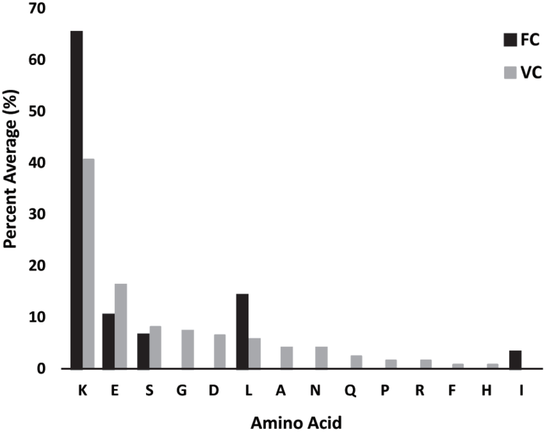

Figure S2 Amino acid distributions in conserved HPRs. Frequencies of amino acids in HPRs conserved across the CVK species (FC) and across Pv strains (VC) are shown. CVK: Cynomolgi-Vivax-Knowlesi group; FC: full conservation; VC: Vivax-conservation.

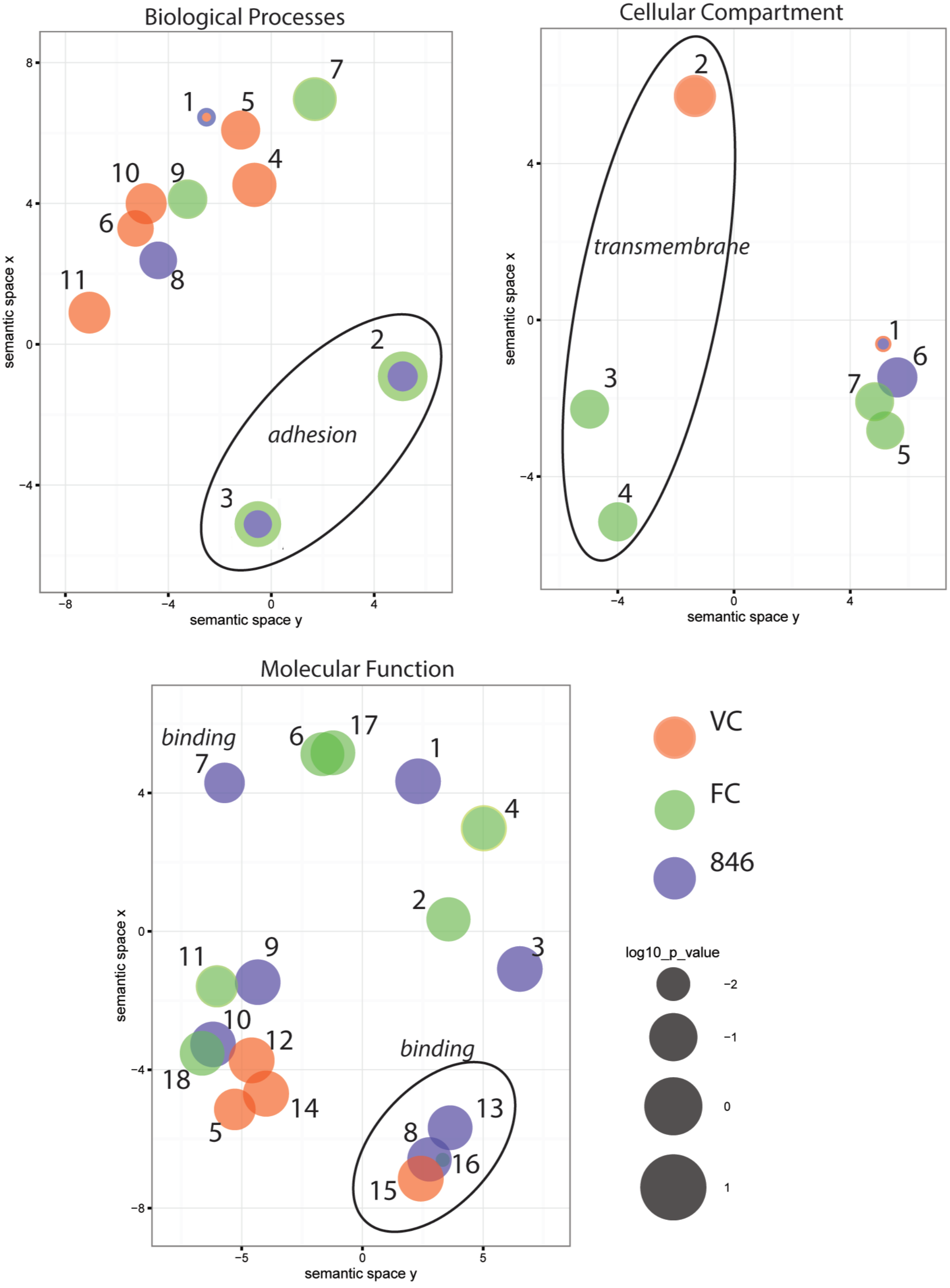

Figure S3 Semantic similarity of gene ontology categories in genes containing HPRs. Gene ontology categories for biological processes, cellular compartment, and molecular function are clustered based on semantic similarity. All genes with at least one HPR (846), those conserved for all CVK species (FC) and those conserved within Pv strains (VC) are shown in blue, green, and red respectively. Circle sizes are proportional to log 10 p-values. Numbers refer to GO IDs from Supplementary Table S1. HPR: homopolymeric regions; CVK: Cynomolgi-Vivax-Knowlesi; FC: full conservation; VC: Vivax-conservation

Supplementary Table S1 List of GO IDs enriched in the 846, VC, and FC gene group. Grey rows are GO IDs not represented in the Revigo plots (Supplementary Fig. S3) because redundant (these categories are represented by the GO ID in the row above). Explanation of the columns is as follows: "Frequency" shows the proportion of each term in the protein annotation database; "Uniqueness" measures if the term is an outlier compared semantically to the rest of the list; "Dispensability" is the semantic similarity threshold at which the term was removed from the list and assigned to a cluster.

GO category	#	Term ID	Description	Frequency	log10 p-value	Uniqueness	Dispensability	Gene group
Biological Process	1	GO:0007017	microtubule-based process	0.04%	-2.5042	0.502	0	846, VC
	2	GO:0007155	cell adhesion	6.44%	-1.6261	0.92	0	846, FC
	3	GO:0022610	biological adhesion	6.44%	-1.543	0.92	0	846, FC
	4	GO:0006928	cellular component movement	0.02%	-1.5046	0.522	0.28	VC
	5	GO:0007018	microtubule-based movement	0.02%	-1.8344	0.522	0.28	VC
	6	GO:0006468	protein phosphorylation	0.24%	-1.9796	0.267	0.307	VC
		GO:0070647	protein modification by small protein conjugation or removal	0.05%	-1.4784	0.327	0.781	846
		GO:0070646	protein modification by small protein removal	0.05%	-1.6928	0.313	1	VC
		GO:0016579	protein deubiquitination	0.05%	-1.6928	0.313	0.781	VC
		GO:0006259	DNA metabolic process	0.29%	-1.3111	0.254	0.74	VC
	7	GO:0006836	neurotransmitter transport	0.78%	-1.467	0.683	0.358	FC
	8	GO:0019219	regulation of nucleobase-containing compound metabolic process	0.07%	-1.9182	0.234	0.367	846
		GO:2001141	regulation of RNA biosynthetic process	0.05%	-1.8186	0.165	0.999	846
		GO:0051056	regulation of small GTPase mediated signal transduction	0.18%	-1.3949	0.265	1.133	VC
		GO:0031323	regulation of cellular metabolic process	0.13%	-1.4754	0.243	0.928	846
		GO:0006355	regulation of transcription, DNA-templated	0.05%	-1.8186	0.165	0.98	846
		GO:0051252	regulation of RNA metabolic process	0.05%	-1.8186	0.179	0.98	846
		GO:0046578	regulation of Ras protein signal transduction	0.18%	-1.3949	0.265	0.757	VC
		GO:0051171	regulation of nitrogen compound metabolic process	0.07%	-1.9182	0.305	0.888	846
	9	GO:0046068	cGMP metabolic process	0.66%	-1.7644	0.219	0.524	FC
		GO:0006182	cGMP biosynthetic process	0.04%	-1.7644	0.302	1.205	FC

		GO:0009190	cyclic nucleotide biosynthetic process	0.04%	-1.3719	0.308	1.215	FC
	10	GO:0016310	phosphorylation	0.62%	-1.7127	0.372	0.574	VC
	11	GO:0043412	macromolecule modification	0.47%	-1.6652	0.429	0.602	VC
Molecular Function	1	GO:0001071	nucleic acid binding transcription factor activity	0.02%	-1.5203	0.937	0	846
	2	GO:0003674	molecular_function	100.00%	-1.4306	1	0	FC
	3	GO:0003700	sequence-specific DNA binding transcription factor activity	0.02%	-1.5203	0.937	0	846
	4	GO:0003735	structural constituent of ribosome	0.08%	-1.3955	0.937	0	FC
	5	GO:0004674	protein serine/threonine kinase activity	0.22%	-1.8835	0.49	0	VC
		GO:0003872	6-phosphofructokinase activity	0.01%	-1.5901	0.537	1.134	FC
		GO:0019200	carbohydrate kinase activity	0.01%	-1.467	0.567	0.703	FC
		GO:0004672	protein kinase activity	0.26%	-2.0715	0.487	0.956	VC
		GO:0008443	phosphofructokinase activity	0.10%	-1.5901	0.478	0.882	FC
		GO:0004713	protein tyrosine kinase activity	0.22%	-1.7562	0.49	0.968	VC
		GO:0016773	phosphotransferase activity, alcohol group as acceptor	0.31%	-1.3657	0.505	0.85	846, VC
	6	GO:0005326	neurotransmitter transporter activity	2.53%	-1.467	0.842	0	FC
	7	GO:0005488	binding	9.46%	-1.9697	0.942	0	846
	8	GO:0005515	protein binding	0.27%	-2.9407	0.833	0	846
	9	GO:0009975	cyclase activity	0.01%	-1.525	0.723	0.228	846
		GO:0019787	small conjugating protein ligase activity	0.02%	-1.3636	0.717	0.236	846
	10	GO:0016849	phosphorus-oxygen lyase activity	0.06%	-1.525	0.678	0.255	846
	11	GO:0008750	NAD(P)+ transhydrogenase (AB-specific) activity	0.07%	-1.7644	0.62	0.259	FC
		GO:0008746	NAD(P)+ transhydrogenase activity	0.07%	-1.7644	0.62	0.999	FC
		GO:0016652	oxidoreductase activity, acting on NAD(P)H, NAD(P) as acceptor	0.07%	-1.7644	0.62	0.999	FC
	12	GO:0008026	ATP-dependent helicase activity	0.24%	-1.4969	0.64	0.287	VC
		GO:0070035	purine NTP-dependent helicase activity	0.24%	-1.4969	0.64	0.915	VC
	13	GO:0005509	calcium ion binding	0.06%	-1.6057	0.845	0.318	846
	14	GO:0003777	microtubule motor activity	0.02%	-1.4758	0.7	0.372	VC

	15	GO:0003677	DNA binding	0.60%	-1.4991	0.824	0.388	VC
	16	GO:0000166	nucleotide binding	7.55%	-1.6391	0.8	0.588	FC
		GO:0036094	small molecule binding	7.57%	-1.5831	0.802	0.827	FC
	17	GO:0015081	sodium ion transmembrane transporter activity	0.38%	-1.3719	0.745	0.597	FC
		GO:0005328	neurotransmitter:sodium symporter activity	0.06%	-1.467	0.779	1.148	FC
		GO:0015370	solute:sodium symporter activity	0.06%	-1.467	0.779	1.148	FC
		GO:0004842	ubiquitin-protein transferase activity	0.35%	-1.4784	0.557	0.646	846
	18	GO:0004383	guanylate cyclase activity	0.01%	-1.3719	0.699	0.65	FC
Cellular Component	1	GO:0015630	microtubule cytoskeleton	0.05%	-2.3646	0.131	0	846, VC
		GO:0000922	spindle pole	0.03%	-1.4458	0.132	0.976	846
		GO:0044430	cytoskeletal part	0.08%	-1.8541	0.117	0.932	846, VC
		GO:0005875	microtubule associated complex	0.02%	-1.3126	0.112	0.951	VC
		GO:0005819	spindle	0.05%	-1.4458	0.116	0.941	846, VC
		GO:0005856	cytoskeleton	0.10%	-1.4709	0.139	0.816	846, VC
	2	GO:0016020	membrane	93.69%	-1.3384	0.99	0	VC
	3	GO:0016021	integral component of membrane	75.63%	-1.4135	0.927	0	FC
	4	GO:0031224	intrinsic component of membrane	75.64%	-1.3955	0.927	0.242	FC
	5	GO:0005945	6-phosphofructokinase complex	0.03%	-1.5901	0.221	0.489	FC
	6	GO:0005634	nucleus	0.37%	-1.4994	0.209	0.607	846
	7	GO:0030529	ribonucleoprotein complex	0.11%	-1.529	0.201	0.698	FC
		GO:0005840	ribosome	0.07%	-1.3955	0.105	0.924	FC
		GO:0030684	preribosome	0.05%	-1.525	0.203	0.728	846
